# Integrating metagenomics and metatranscriptomics into Orthoflavivirus diagnosis: a transformative approach for clinical virology

**DOI:** 10.1099/jgv.0.002247

**Published:** 2026-03-30

**Authors:** Md. Eram Hosen, Paul F. Horwood, Subir Sarker

**Affiliations:** 1 Biomedical Sciences and Molecular Biology, College of Medicine and Dentistry, James Cook University, Townsville, QLD, 4811, Australia; 2 Australian Institute of Tropical Health and Medicine, James Cook University, Townsville, QLD 4811, Australia; 3 College of Science and Engineering, James Cook University, Cairns, QLD, 4811, Australia; 4 Australian Institute of Tropical Health and Medicine, James Cook University, Cairns, QLD, 4811, Australia; 5 Department of Microbiology, Anatomy, Physiology and Pharmacology, School of Agriculture, Biomedicine and Environment, La Trobe University, Melbourne, Victoria, 3086, Australia

**Keywords:** conventional methods, global implementation, metagenomic, metatranscriptomics, *Orthoflavivirus *diagnosis

## Abstract

Diagnostic inaccuracies are a major yet often overlooked threat to global health, leading to delayed treatment, preventable harm and systemic gaps in disease control. Among the most affected domains are *Orthoflavivirus* infections, which pose ongoing diagnostic challenges due to antigenic cross-reactivity, overlapping clinical symptoms and the narrow temporal sensitivity of standard tools such as serology and reverse transcription polymerase chain reaction. These constraints have led to widespread misdiagnoses and underreporting, ultimately hampering both effective clinical management and public health response. Recent advances in metagenomic and metatranscriptomic sequencing offer a transformative solution by enabling unbiased, simultaneous pathogen detection and real-time profiling of viral and host transcriptomics. In this review, we assess the diagnostic performance and translational value of these approaches in resolving *Orthoflavivirus* infections, with case examples from clinical settings in countries like the USA, UK, China and Germany which have already implemented these approaches into routine diagnosis in some settings. We examine key methodological considerations, including optimal sample timing, sample types and processing, sequencing strategy selection and the diagnostic performance of various platforms. We highlight the growing use of metatranscriptomics for detecting active infections, profiling viral and host responses, identifying coinfections and supporting real-time surveillance. We also discuss the key challenges such as technical expertise, lack of standardization, cost, turnaround time and regulatory approval that currently limit global implementation. Finally, we highlight emerging international efforts to integrate sequencing-based diagnostics into routine hospital workflows. Together, these innovations mark a critical shift toward precision diagnostics for *Orthoflavivirus* infections, with broad implications for clinical settings.

## Introduction

Orthoflaviviruses (formerly classified under the genus *Flavivirus*), including dengue virus (DENV), Zika virus (ZIKV), Japanese encephalitis virus (JEV), yellow fever virus (YFV) and West Nile virus (WNV), pose significant public health threats across tropical and subtropical regions, with global expansion fuelled by climate change, urbanization and increased human mobility [1,5]. Despite their growing burden, accurate and timely diagnosis of these infections remains a critical challenge in both endemic and non-endemic regions [6,7]. Clinical manifestations of these infections are often nonspecific and overlapping, and their frequent co-circulation complicates diagnosis [6]. Traditional diagnostic tools, such as serological assays and PCR-based methods, are the gold standard for early clinical detection of known viruses; however, they suffer from important limitations, including cross-reactivity, narrow detection windows and the need for prior pathogen knowledge that frequently led to underdiagnosis, misclassification or delayed detection [7,10]. In this context, viral metagenomic and metatranscriptomic sequencing approaches represent a paradigm shift in infectious disease diagnostics. These high-throughput, untargeted technologies enable comprehensive profiling of the virome or viral transcriptome in clinical and environmental samples, without requiring prior assumptions about the pathogen [11,14]. By simultaneously identifying known, novel and co-infecting viruses, including low-abundance or divergent variants, these methods offer a powerful alternative to the linear logic of conventional diagnostics [11,15, 16]. In addition to enhancing sensitivity and specificity, metagenomics and metatranscriptomics provide genomic insights into viral evolution, virulence, transmission patterns and drug resistance, facilitating real-time epidemiological surveillance and precision medicine [11,17, 18].

Various institutions and hospitals in the USA, UK, France, Germany, South Korea, Japan and China have already implemented metagenomic and metatranscriptomic sequencing approaches in clinical settings for the detection of viruses [19,24]. These pioneering programmes underscore the clinical viability of these technologies, particularly in diagnosing atypical or unexplained viral infections where conventional diagnostics fail. Integration into routine diagnostics is becoming increasingly feasible, driven by advancements in sequencing technologies, streamlined library preparation and clinician-accessible bioinformatics platforms. Nevertheless, broad adoption remains limited, particularly in low-resource, high-burden regions, where orthoflaviviruses are most endemic. Bridging this gap will require targeted investment in infrastructure, training and equitable access to ensure global clinical impact.

This review critically examines the current state-of-the-art approaches in viral metagenomics and metatranscriptomics for *Orthoflavivirus* detection, evaluates their clinical applicability and diagnostic advantages and discusses barriers to implementation followed by future directions. We propose that integrating these approaches into the clinical diagnostic landscape will not only overcome the shortcomings of conventional methods but also redefine how *Orthoflavivirus* infections are detected, monitored and managed in the twenty-first century.

## Conventional clinical diagnostic methods for *Orthoflavivirus* detection

**Fig. 1.**
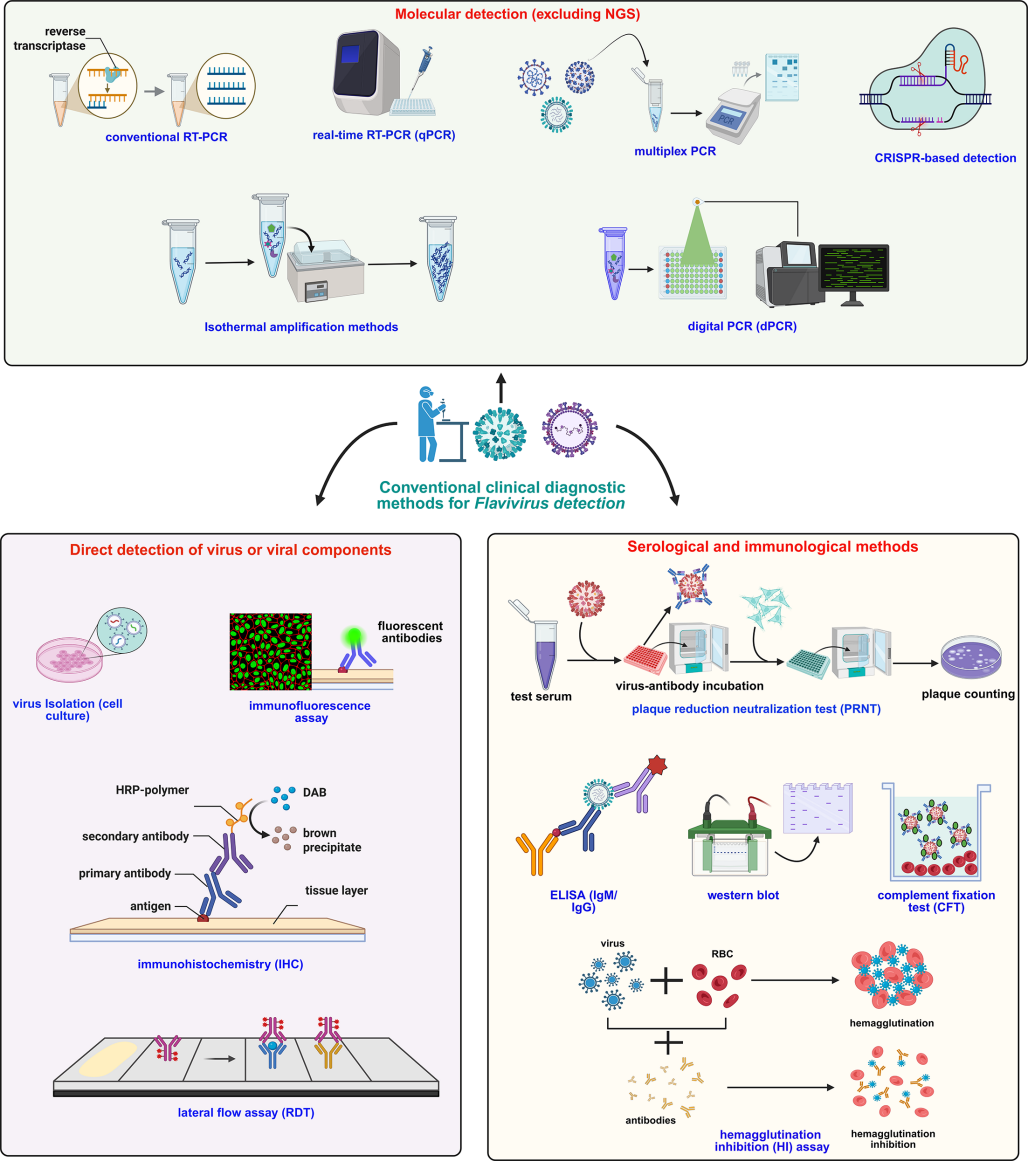
Overview of conventional methods for *Orthoflavivirus* detection. The detection approaches are broadly categorized into three groups: direct detection, molecular diagnostics and serological or immunological assays. Direct detection methods include virus isolation, IHC, rapid antigen tests and IFA, which identify viral particles or antigens directly from clinical or vector samples. Molecular diagnostics encompass nucleic acid-based techniques such as conventional PCR, reverse transcription polymerase chain reaction (RT-PCR), multiplex PCR, isothermal amplification methods, digital PCR (dPCR) and CRISPR-based assays, enabling sensitive and specific detection of viral RNA. Serological or immunological assays such as plaque reduction neutralization test (PRNT), enzyme-linked immunosorbent assay (ELISA), blotting techniques, complement fixation test (CFT) and haemagglutination inhibition (HI) detect host antibody responses or viral antigens, aiding in diagnosis and epidemiological surveillance. Created with BioRender (https://www.biorender.com/).

Viral detection is typically feasible during the early acute phase of illness, while antibody-based methods, primarily IgM and occasionally IgG, are more effective a few days post-infection. Optimal diagnosis often combines viral RNA or antigen detection with serological assays, with test selection influenced by clinical objectives, surveillance needs and practical considerations such as local resource availability, laboratory capacity and technical capability [7]. *Orthoflavivirus* infection diagnosed by conventional clinical diagnostic approaches can be broadly classified into three categories: direct detection, molecular diagnostics and serological or immunological assays (Fig. 1). Direct detection methods such as virus isolation, immunohistochemistry (IHC), rapid antigen tests and immunofluorescence assays (IFAs) involve identifying viral particles or antigens in clinical samples. Virus isolation using cell culture is the primary clinical method for confirming live orthoflaviviruses, supporting phenotypic and antiviral studies [25]. However, its clinical diagnostic use is limited by time, labour, biosafety requirements and reduced sensitivity in late or low-viraemia cases. Moreover, orthoflaviviruses differ in replication efficiency across mosquito- and mammalian-derived cell lines. For instance, DENV and ZIKV replicate efficiently in both C6/36 *Aedes albopictus* mosquito cells and mammalian cell lines such as African green monkey kidney cells (Vero) [26,28], whereas WNV, JEV and YFV are commonly propagated in Vero, baby hamster kidney 21 cells (BHK-21) or related kidney-derived cell lines [25,29,31]. These differences in replication kinetics and viral yield necessitate careful selection of cell culture systems to optimize virus recovery and detection. Recent studies have demonstrated that IFA enables highly sensitive detection of intracellular flaviviral antigens, such as E and NS1, directly from cell culture samples within ~1 h [32,34], whereas IHC can identify flaviviral antigens within tissue sections, providing valuable pathological context, especially in biopsies or post-mortem samples, though it is less rapid and requires tissue processing [35,37]. In contrast, lateral flow immunoassays rapidly (15–30 min) recognize flaviviral antigens in blood or serum, facilitating user-friendly, point-of-care early diagnosis in field or clinical settings [38,39]. However, these methods have notable limitations in clinical diagnosis, particularly in cases with low viral load, unknown antigen distribution or during the later stages of infection. Additionally, they are typically pathogen-specific and may not reliably detect co-infections, as they often fail to identify multiple viruses present in the same sample [9,32, 36, 38, 40] (Fig. 1 and Table 1).

**Table 1.** Overview of conventional clinical diagnosis methods for *Orthoflavivirus* detection

Category	Method	Advantages	Disadvantages/limitations
Direct detection	Virus isolation	Confirming live virus Enables downstream studies Specificity	Time-consuming Cell culture adaptation risk Requires BSL-2/3 facilities Low sensitivity Require specificity cell lines
	IHC	Tissue-based detection Post-mortem or biopsy	Low sensitivity in early infection Cross-reactivity possible
	IFA	Cell/tissue-based antigen detection Confirmatory	Requires fluorescence microscopy and expertise Interpretation subjectivity Low sensitivity
	RDT	Rapid results (<30 min) Field- and resource-friendly Easy-to-use and low-cost	Lower sensitivity Prone to false negatives Limited availability for some flaviviruses
Molecular diagnostics	Conventional RT-PCR	High sensitivity Specificity and sensitivity Early detection	Cannot detect unknown viruses Cross-reactivity False-positive/false-negative results Misdiagnosis Limited in detecting co-infections
	Real-time RT-PCR (qRT-PCR)	Quantitative High specificity Acute-phase detection Specificity and sensitivity	Sensitive to RNA degradation Equipment/trained personnel needed False-positive/false-negative results Misdiagnosis Limited in detecting co-infections
	Multiplex PCR	Detects multiple pathogens simultaneously Useful in co-infection settings Specificity and sensitivity	Primer interference possible Complex optimization Potential cross-reactivity False-positive/false-negative results Misdiagnosis
	LAMP	Rapid and simple Field-deployable Specificity and sensitivity	Limited non-specific amplification Potential cross-reactivity False-positive/false-negative results Misdiagnosis
Serological/immunological assays	IgM ELISA	Detects recent infection Widely available Easy to perform	Cross-reactivity IgM may persist for months Cannot distinguish primary vs secondary infection False-positive/false-negative results Misdiagnosis
	IgG ELISA	Indicates past exposure or immunity Useful for surveillance	Cross-reactivity Not useful for acute diagnosis False-positive/false-negative results Misdiagnosis
	Western blot	High specificity for detecting viral proteins or antibodies Can identify specific viral antigens or antibody isotypes	Technically complex and time-consuming Not standardized for routine clinical use Expensive and low throughput Cross-reactivity
	CFT	Detects antigen–antibody complexes via complement consumption	Low sensitivity, especially in early or mild infection Poor specificity and outdated Cannot differentiate between past and recent infections
	HI	Historically used in surveillance Detects antibodies	Poor specificity Obsolete in modern clinical settings Labour-intensive
	PRNT	Serotype-specific antibody detection Resolves cross-reactivity	Laborious and time-consuming Requires BSL-2/3 lab Not routine in clinical settings False-positive/false-negative results Misdiagnosis

CFT, complement fixation test; ELISA, enzyme-linked immunosorbent assay; HI, haemagglutination inhibition; IFA, immunofluorescence assay; IHC, immunohistochemistry; LAMP, loop-mediated isothermal amplification; PRNT, plaque reduction neutralization test; RDT, rapid diagnostic test.

RT-PCR is widely employed for the clinical detection of DENV, ZIKV, YFV, WNV and other *Orthoflavivirus* due to its high sensitivity and specificity during the early viraemic phase [7,41, 42]. In recent years, RT-PCR has been used for simultaneous detection of DENV and ZIKV, often alongside chikungunya virus (CHIKV), an alphavirus that co-circulates with orthoflaviviruses in many endemic regions. Such approaches have been applied in outbreak investigations and routine diagnostics, enabling rapid and accurate viral identification in both endemic and emerging settings [7,41, 43]. In China, RT-PCR has enabled detection of JEV alongside six other flaviviruses [44,45] and has also supported mosquito-based surveillance of DENV and CHIKV in Africa [46]. RT-PCR has been adopted in clinical diagnostics for the simultaneous detection of co-circulating known flaviviruses. For example, it has been used to detect all four DENV serotypes, along with ZIKV and CHIKV RNA, during the 2020–2022 outbreaks in Vietnam [47,48]. Similar applications include detection of ZIKV and DENV co-infections in Brazil and Bangladesh [49,50]. Beyond routine clinical diagnostics, multiplex PCR assays have also been evaluated using animal-origin samples and reference materials from Sub-Saharan Africa, including Rift Valley fever virus, to demonstrate assay performance [51], and have been applied to ecological surveillance, including detection of multiple viruses in bat populations in Australia [52]. While PCR-based surveillance remains highly sensitive and specific for known viral targets, it is inherently limited to the sequences it is designed to detect. In contrast, next-generation sequencing offers the unique advantage of identifying novel or unexpected viral strains, although it may face challenges with sensitivity and contamination. Similarly, isothermal amplification methods, such as loop-mediated isothermal amplification and recombinase polymerase amplification, can detect flaviviral RNA within 30 min with high sensitivity and specificity. However, these methods are limited by their reduced multiplexing capabilities and a higher risk of non-specific amplification [53,54]. Emerging approaches like CRISPR-based diagnostics (e.g. SHERLOCK and DETECTR) are redefining point-of-care testing with high specificity and portability, though widespread clinical implementation is still evolving [55]. dPCR enables highly sensitive and accurate detection of known flaviviruses with absolute quantification and monitoring in low viral load samples but is limited to targeting single, known viruses and requires specialized equipment, making it less suitable for broad or unknown virus screening [56,57] (Fig. 1 and Table 1).

Serological assays remain crucial in flavivirus clinical diagnosis, especially when direct viral detection is limited to early infection [10,58]. Among these methods, ELISAs that detect virus-specific IgM and IgG antibodies are widely used in the clinical sector due to their scalability and cost-effectiveness [58,60]. IgM antibodies serve as reliable clinical diagnostic markers for early infection, typically emerging during the acute and early convalescent phases, whereas IgG responses indicate prior exposure and long-term immune memory, providing critical insights into both individual infection history and population-level immunity [61,63]. However, serological cross-reactivity among flaviviruses, particularly DENV and ZIKV, poses significant diagnostic challenges [59,64, 65]. The PRNT can address these limitations by confirming neutralizing antibodies in cases of cross-reactivity, providing quantitative titres that are essential for vaccine evaluation and epidemiological studies [59,65]. Similarly, Western blot is primarily used as a confirmatory assay due to its high specificity in detecting antibodies against distinct flaviviral proteins such as envelope (E) and non-structural protein 1 (NS1). This technique helps resolve cross-reactivity between closely related flaviviruses like DENV, JEV and ZIKV, particularly in secondary infections [66,67]. In contrast, CFT, one of the earliest serological methods employed to detect antibodies against flaviviruses, is now largely obsolete due to its low sensitivity and inability to differentiate closely related viruses [68,69]. Complementary assays like HI and microneutralization (MN) remain useful in flavivirus diagnosis, with HI providing cost-effective broad antibody detection despite cross-reactivity, while MN offers greater specificity and sensitivity for distinguishing closely related viruses such as dengue and Zika [7,10, 70] (Fig. 1 and Table 1).

Despite their widespread application for *Orthoflavivirus* diagnosis, conventional diagnostic techniques suffer from important limitations, including cross-reactivity, narrow detection windows and a reliance on prior pathogen knowledge. These constraints frequently lead to false-positive or false-negative results, misclassification or delayed detection, particularly in settings where multiple orthoflaviviruses co-circulate or novel variants emerge. Moreover, the inability of these methods to detect unexpected or co-infecting pathogens hampers accurate clinical management and timely outbreak response.

## Limitations of current *Orthoflavivirus* diagnostic methods: a public health concern

Misdiagnosis of *Orthoflavivirus* infections through conventional diagnostic methods represents a significant challenge to both clinical management and public health systems globally. Misdiagnosis or delayed diagnosis can lead to severe complications, such as organ failure, neurological damage or death, especially in vulnerable populations [71]. Moreover, diagnostic inaccuracies undermine surveillance and outbreak control, allowing viruses to spread unchecked, causing inefficient use of healthcare and public health resources. Although precise global mortality attributable solely to *Orthoflavivirus* diagnostic limitation and their public health implications remains poorly quantified. However, some documented cases illustrate these consequences. *Orthoflavivirus* diagnostic limitations have caused severe outcomes, including fatal DENV encephalitis in India [72], paralysis from WNV misdiagnosed as stroke in the USA [73] and missed ZIKV cases in Bangladesh [74] that impeded timely public health responses. Such examples highlight the clinical and epidemiological consequences of inadequate or delayed laboratory confirmation. *Orthoflavivirus* infections frequently present with non-specific febrile symptoms that overlap with bacterial, respiratory and other viral diseases, complicating clinical differentiation [7,75, 76]. In endemic settings, the accuracy of clinical diagnosis remains limited. For example, primary healthcare physicians in Honduras reported only 30.8% overall diagnostic accuracy for suspected *Orthoflavivirus* infections, with RT-qPCR confirmation in just 8.2% of dengue and 27.1% of Zika cases, and nearly half (45.5%) of patients meeting multiple *Orthoflavivirus* case definitions simultaneously [77]. Similarly, surveillance datasets from Brazil indicate that only about half of reported DENV and CHIKV cases are laboratory-confirmed due to symptom overlap and limited confirmatory testing, contributing to both under- and over-reporting in this region [78]. These diagnostic inaccuracies can lead to misclassification of infections, affecting surveillance data and consequently guiding inappropriate vaccine strain selection. Such mismatches may induce suboptimal immunity, which, in turn, can impose immune selection pressures that drive the emergence of antigenic variants with enhanced fitness or immune escape potential [79]. This is particularly concerning in the context of orthoflaviviruses like DENV and ZIKV, where shared epitopes and partial cross-immunity can complicate host-pathogen interactions. Thus, improving diagnostic precision is not merely a technical concern but a critical necessity for effective clinical care, immunization strategies and virological risk forecasting. These challenges highlight the critical need for improved diagnostic tools and clinical protocols to reduce the burden of *Orthoflavivirus* misdiagnosis. To address these diagnostic blind spots, metagenomic and metatranscriptomic sequencing have emerged as powerful, unbiased approaches capable of comprehensively characterizing viral diversity in both clinical and environmental samples.

## Viral metagenomic sequencing for *Orthoflavivirus* diagnostics

The advent of viral metagenomic sequencing has revolutionized pathogen detection by enabling unbiased, hypothesis-free identification of viral genomes across clinical and environmental samples [12,13]. In *Orthoflavivirus* endemic regions, where co-circulating viruses such as DENV, ZIKV, JEV and WNV often present with indistinguishable clinical features, this approach offers a critical advantage over traditional diagnostics such as RT-PCR, ELISA or virus isolation, each of which suffers from limited target scope, cross-reactivity and reduced sensitivity in early or asymptomatic infections [16,80, 81]. By capturing a broad spectrum of viral sequences without prior assumptions, metagenomic tools facilitate comprehensive, real-time diagnosis, strain differentiation and surveillance, which is particularly valuable in contexts of ambiguous clinical presentation or unexpected outbreaks.

**Fig. 2.**
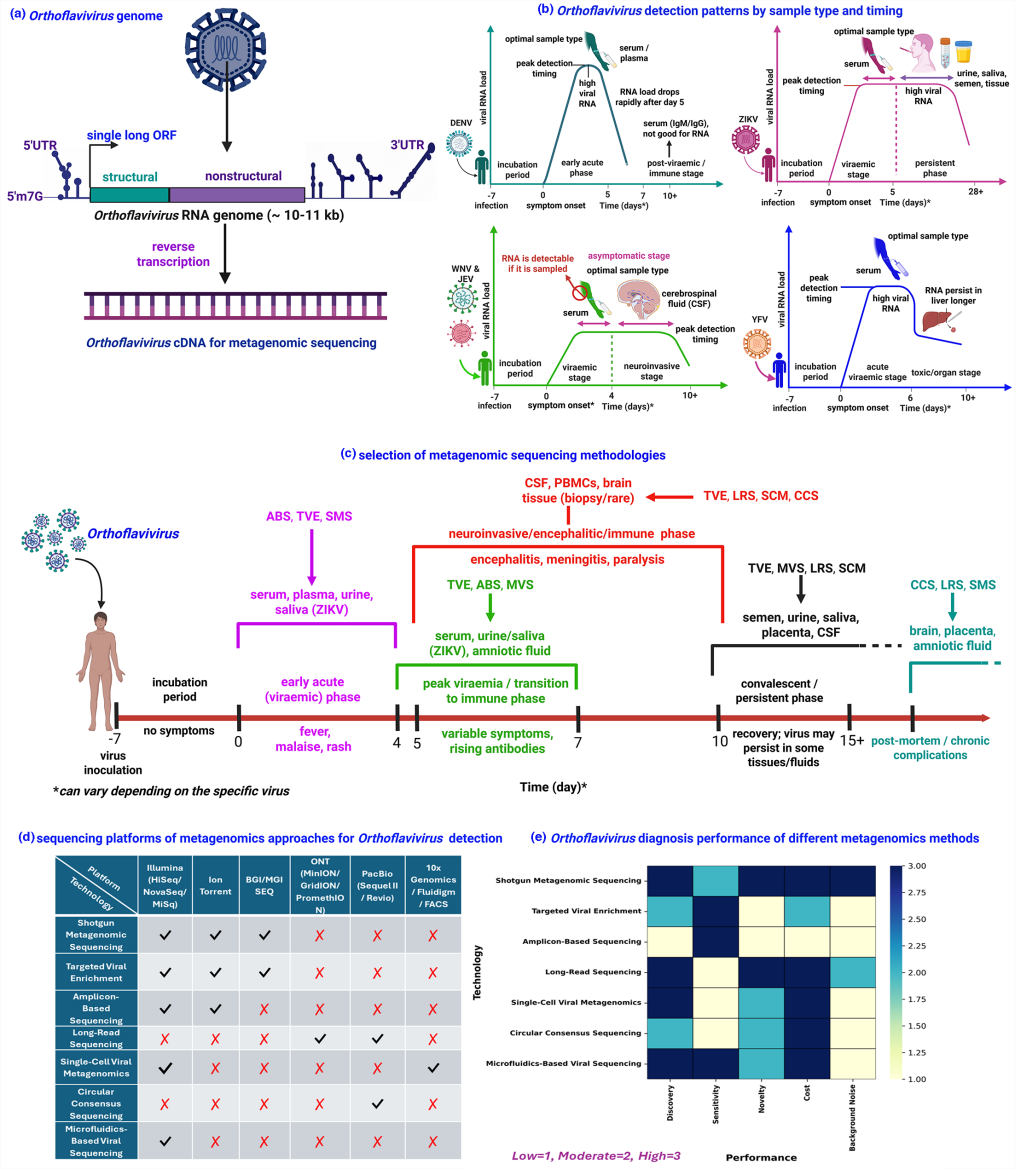
Viral metagenomic sequencing and diagnostic applications for *Orthoflavivirus* detection. (**a**) Schematic representation of the *Orthoflavivirus* genome and the initial steps of metagenomic sequencing, illustrating the requirement for reverse transcription of viral RNA to cDNA due to the RNA nature of *Orthoflavivirus* genomes. (**b**) Detection kinetics of viral RNA for five major orthoflaviviruses such as DENV, ZIKV, WNV, JEV and YFV based on infection time course and sample type, with line graphs showing temporal variation in viral load and sample-specific detection windows. (**c**) Selection matrix for metagenomic methodologies, indicating the optimal sequencing approaches tailored to different sample types and stages of infection. (**d**) Sequencing platforms used in various metagenomic approaches to corresponding library preparation methods and diagnostic workflows. (**e**) Comparative performance heatmap of metagenomic methods, evaluated across five diagnostic criteria to highlight the relative strengths of each approach in *Orthoflavivirus* detection. Heatmap values (low=1, moderate=2, high=3) represent author-assigned, semi-quantitative scores derived directly from the narrative evaluation in the main text, reflecting relative performance across discovery potential, analytical sensitivity, novelty detection, cost and background noise. Scores summarize recurring trends reported across the cited literature and are intended as a conceptual guide rather than a quantitative benchmarking analysis. Created with BioRender (https://www.biorender.com/).

Orthoflaviviruses possess a ~11 kb positive-sense ssRNA genome with a 5′ cap and structured 3′ UTR lacking a poly(A) tail. The genome contains a single ORF encoding structural (C, prM and E) and non-structural (NS1–NS5) proteins [1,79]. Despite all orthoflaviviruses being single-stranded positive-sense RNA viruses, they can be effectively detected using metagenomic approaches when paired with RNA-to-cDNA conversion, thus expanding the utility of metagenomic sequencing beyond DNA viruses (Fig. 2a). Clinical workflows now routinely integrate reverse transcription during nucleic acid extraction, allowing metagenomic libraries to capture viral RNA genomes alongside DNA. This has enabled identification of flaviviruses in febrile illness cases with unknown aetiology, neurological syndromes, congenital infections and co-infection scenarios [82,83]. Recent studies have demonstrated the global impact of viral metagenomic next-generation sequencing (mNGS) in flavivirus detection. A Sri Lankan investigation of febrile RT-PCR–negative patients uncovered several dengue cases and even co-infections misdiagnosed as chikungunya using mNGS [84]. Similar approaches in China, India and Australia have detected DENV in clinical samples that conventional assays failed to detect [85,86]. mNGS techniques have also been successfully applied to identify ZIKV from cerebrospinal fluid (CSF), placental tissues and other clinical specimens, both in regions experiencing outbreaks and in non-epidemic settings [84,87]. Similarly, mNGS has been widely applied globally for the direct clinical detection of WNV, JEV and YFV, particularly in cases where conventional diagnostic methods fail to identify the causative agent [88,90]. Additionally, several recent studies revealed that mNGS can simultaneously detect multiple co-circulating flaviviruses from both human and mosquito (entomological) samples, including previously uncharacterized viral lineages [88,92]. Together, these cases underscore how mNGS transcends conventional diagnostics, enabling hypothesis-free, strain-resolved detection of diverse orthoflaviviruses in both clinical and ecological settings. Collectively, these applications highlight the global clinical utility of metagenomics in diagnosing and monitoring the diverse and co-circulating orthoflaviviruses that challenge conventional diagnostic approaches.

However, the sensitivity of viral metagenomic sequencing for *Orthoflavivirus* detection is critically shaped by several sample-dependent variables, including viral load, timing of collection and the biological matrix used (Fig. 2b). During the early acute phase (typically within days 1–5 post-infection), serum or plasma is the most informative sample for viraemic such as DENV, ZIKV and YFV, where viral RNA levels are transiently high [45,93]. In contrast, neurotropic orthoflaviviruses like WNV and JEV are often no longer detectable in the blood by the time neurological symptoms appear, requiring CSF sampling, although RNA levels in CSF remain low [93,96]. ZIKV presents a unique diagnostic window, with RNA persisting in urine, saliva, semen and tissues, including placenta and brain beyond the acute serum phase, facilitating detection well into convalescence or in congenital cases [97,98] (Fig. 2b). Tick-borne encephalitis virus (TBEV), which follows a biphasic course, may be detected in serum during the initial febrile phase (days 1–4), whereas CSF becomes critical during the second, neuroinvasive phase [99,100]. Across orthoflaviviruses, optimal detection requires aligning sampling strategy with viral kinetics, yet even then, low viral loads, short viraemia and sample degradation can limit sensitivity, necessitating the integration of appropriate metagenomic approaches [9].

To address these challenges, careful selection of metagenomic sequencing methodologies is required which include shotgun metagenomic sequencing (SMS), targeted viral enrichment (TVE), amplicon-based sequencing (ABS), long-read sequencing (LRS), single-cell viral metagenomics (SCM), circular consensus sequencing (CCS) and microfluidics-based viral sequencing (MVS). Each platform is tailored to address distinct diagnostic challenges ranging from broad-spectrum virus detection [101] and low-abundance genome recovery to high-resolution genome reconstruction [102,103], co-infection profiling [104] and virus–host interaction analysis [105,106]. In the early acute phase of infection, serum or plasma is the preferred sample type for detecting viraemic orthoflaviviruses, where methods such as ABS, TVE or SMS are well suited for capturing the brief window of elevated viral RNA levels [84,107, 108]. As the infection progresses (days 4–7), the viral RNA may persist in urine, saliva and amniotic fluid, favouring continued detection through TVE, ABS or MVS [107,109, 110]. In neurotropic orthoflaviviruses such as WNV, JEV or TBEV, the neuroinvasive phase (typically days 5–10 or later) necessitates CSF sampling, though viral loads are often low, requiring high-sensitivity methods such as TVE, CCS, SCM or LRS [88,111,115]. In late-stage or fatal cases, viral RNA may persist in brain or placental tissues, where CCS and LRS provide the resolution needed for genomic characterization [116] (Fig. 2c). Across all stages, method selection must account for both sample type and viral load, as detection sensitivity hinges on aligning sequencing strategies with the kinetics and tissue tropism of each *Orthoflavivirus*.

### Choosing metagenomic sequencing platforms for *Orthoflavivirus* diagnosis

The effectiveness of metagenomic diagnostics for orthoflaviviruses is closely linked to sequencing platform selection. A comparative matrix analysis illustrating the compatibility of metagenomic approaches with various sequencing platforms is shown in Fig. 2(d). For high-throughput detection of orthoflaviviruses, short-read platforms like Illumina (e.g. MiSeq and NovaSeq), Ion Torrent and BGI/MGI platforms are standard for SMS and TVE sequencing, offering high accuracy for detecting co-infections and resolving viral genotypes or lineages [117,119]. Similarly, ABS is best suited to Illumina and Ion Torrent platforms due to their efficiency in handling short DNA fragments [120,121], while Oxford Nanopore Technologies and PacBio are preferred for long-read RNA sequencing for whole-genome reconstruction and structural variant detection, particularly in recombinant or segmented flaviviruses [111,122]. Similarly, CCS also uses PacBio to generate high-fidelity reads, aiding in resolving closely related serotypes and minor variants [123,125]. Single-cell and microfluidics-based metagenomics, though emerging, utilize platforms like 10x Genomics and Fluidigm for cell sorting or droplet barcoding, with sequencing performed on Illumina systems. These approaches enable rare variant detection and virus–host interaction mapping at single-cell resolution [126,129]. Moreover, the performance of each mNGS approach underscores how diverse sequencing technologies align with key diagnostic goals for flavivirus pathogens (Fig. 2e). To facilitate comparison across sequencing platforms, qualitative descriptors used throughout the text (e.g. high discovery potential and moderate sensitivity) were mapped to a simple ordinal scale (1–3) and visualized as a heatmap (Fig. 2e). This figure provides a visual synthesis of the narrative assessment, with performance categories (low, moderate and high) assigned based on the comparative strengths and limitations discussed for each metagenomic approach, rather than derived from independent benchmarking experiments. Technologies such as SMS offer high discovery potential and detection of novel pathogens, making it ideal for detecting unknown flaviviruses; however, this approach is associated with high costs and significant background noise that may complicate data interpretation [119,130,132]. For routine diagnostic applications, TVE and ABS enhance sensitivity, reduce background noise and provide cost-effectiveness. However, these approaches have limited capacity for detecting novel or highly divergent viral sequences due to their dependence on predefined probe or primer sets, making them well-suited for known viruses and outbreak surveillance rather than pathogen discovery [133,138]. Conversely, LRS enables full-genome assembly with high potential for novel pathogen discovery, but it is limited by low sensitivity and high cost [111,139]. Emerging platforms like SCM and MVS represent the next frontier in *Orthoflavivirus* diagnostics, offering ultra-sensitive, cell-level resolution of infection dynamics and enabling high-throughput diagnostics with reduced reagent use (Fig. 2e) [113,140,144]. This comparative landscape facilitates the alignment of technological capabilities with clinical and epidemiological needs, thereby advancing precision diagnostics of orthoflaviviruses in endemic and epidemic settings.

### Why should mNGS be implemented in clinical settings?

The unbiased, high-throughput capabilities of mNGS have substantially transformed viral diagnostics, particularly in the identification of misdiagnosed or undetected flavivirus infections. In clinical contexts where conventional diagnostics such as serology, PCR or culture may yield inconclusive results or fail entirely, mNGS offers a comprehensive, untargeted alternative capable of revealing both known and unknown pathogens directly from clinical samples, with appropriate sample processing. A striking example of the diagnostic power of mNGS involved the detection of DENV-1 in a respiratory specimen from a febrile patient initially shown to be negative by conventional molecular assays [145]. This corrected diagnosis challenged the assumption that DENV is strictly bloodborne, highlighting mNGS’s ability to detect *Orthoflavivirus* RNA in atypical clinical samples, a critical advantage when investigating outbreaks or evaluating returned travellers. Another example from a Lassa fever outbreak highlights both the challenges and strengths of sequencing approaches: initial Nanopore sequencing misclassified a sample as hepatitis A virus, but subsequent shotgun mNGS provided near-complete coverage of Lassa virus RNA, demonstrating the value of complementary sequencing methods [146]. Although Lassa virus is not an *Orthoflavivirus*, showcasing its value in resolving diagnostic ambiguity in co-endemic regions. Similarly, in a 2012 paediatric gastroenteritis outbreak in New Zealand, mNGS identified human parechovirus 3, which clinically resembles ZIKV in neonates, after standard qPCR failed, thus emphasizing the importance of broad-spectrum tools in differentiating symptomatically overlapping infections [147]. Perhaps the most compelling evidence for mNGS lies in its use for central nervous system (CNS) infections, exemplified by a fatal case of St. Louis encephalitis virus infection in California that was diagnosed only through unbiased mNGS of CSF after negative serology and PCR [148]. Similarly, chronic DENV encephalitis in a 20-year-old male was detected via mNGS of brain biopsy tissue after exhaustive conventional testing failed [149]. These examples show how mNGS can overcome challenges like transient viraemia, immune complex interference or low viral loads in the CNS. The prospective study by Piantadosi *et al*. [150] further demonstrates mNGS’s value in CNS infections, where mNGS of CSF from 68 patients uncovered hard-to-detect pathogens like Powassan virus and *Borrelia burgdorferi*, missed by standard tests, thus highlighting mNGS’s value in detecting rare or low-abundance orthoflaviviruses in CNS infections [150]. Large-scale surveillance data further support mNGS’s clinical utility; for instance, a study in Uganda applied mNGS to 230 undiagnosed febrile cases, uncovering missed DENV-1, DENV-3 and YFV infections, highlighting mNGS’s value in serotype-level detection and outbreak preparedness in endemic regions [151]. A systematic review by Fong *et al*. (2022) found that only 34.7% of purported DENV encephalitis cases met stringent criteria for ‘definite’ or ‘probable’ diagnosis, largely due to ambiguous serological results and overlapping clinical features [152]. These findings advocate for integrating mNGS as a confirmatory tool in the diagnostic workflow of *Orthoflavivirus*-associated encephalitis. Although not exclusive to orthoflaviviruses, numerous studies have reported mNGS outperforming conventional assays across a range of viral infections. In pulmonary infections, Huang *et al*. reported an 88.3% viral detection rate using mNGS, far surpassing PCR [153]. Similarly, Fang *et al*. [154] and Wang *et al*. [155] showed that mNGS identified pathogenic viruses like cytomegalovirus and bocavirus in PCR-negative cases, while Xie *et al*. [156] detected *Pneumocystis jirovecii* co-infection in HIV-positive patients undiagnosed by conventional methods. These findings reinforce mNGS’s role in correcting diagnostic blind spots, a utility directly extendable to *Orthoflavivirus* infections that share clinical overlaps with numerous other pathogens.

## Viral metatranscriptomics for *Orthoflavivirus* detection

**Fig. 3.**
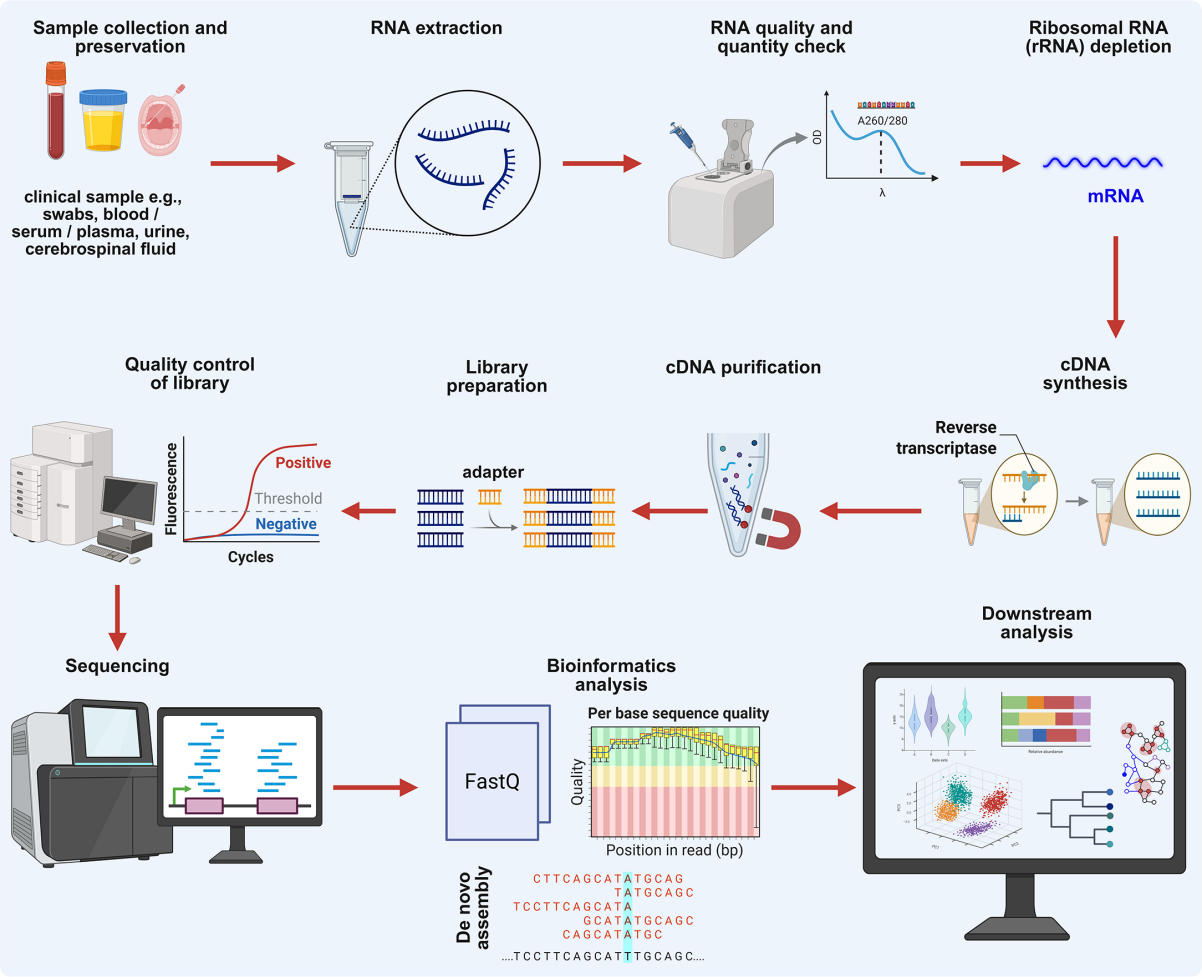
Workflow of viral metatranscriptomic sequencing for *Orthoflavivirus* detection. The metatranscriptomic sequencing process used to detect and characterize orthoflaviviruses directly from clinical samples. The workflow begins with total RNA extraction, followed by rRNA depletion or host RNA subtraction to enrich for viral transcripts. This is followed by reverse transcription to generate cDNA, library preparation and high-throughput sequencing. Bioinformatic analysis pipelines are then applied to filter, assemble and annotate viral transcripts, enabling detection of known and novel orthoflaviviruses, quantification of viral gene expression and simultaneous profiling of host immune responses. Created with BioRender (https://www.biorender.com/).

Another approach to overcome the *Orthoflavivirus* diagnostic limitation of conventional methods is viral metatranscriptomics. The comprehensive sequencing of total RNA within a biological sample has emerged as a powerful tool for the detection and characterization of RNA viruses. Unlike metagenomics, which identifies the genetic material of all organisms present (DNA and RNA), metatranscriptomics captures the actively expressed genes, offering a dynamic snapshot of infection and host–pathogen interactions. Given that most arboviruses, and all orthoflaviviruses, are ssRNA viruses, it is uniquely suited to detect their genomic and sub-genomic RNA during active replication. This technique enables not only the identification of known and novel orthoflaviviruses but also differentiation between latent and replicating viral infections, which is an advantage over DNA-based metagenomics, making it an invaluable tool in clinical diagnostics. As shown in Fig. 3, a typical metatranscriptomic workflow involves total RNA extraction from clinical matrices (e.g. blood, serum, urine and CSF), followed by rRNA depletion, library preparation, deep sequencing typically carried out on Illumina platforms generating paired-end reads and comprehensive bioinformatics analysis. The removal of host reads allows for sensitive detection of viral transcripts and co-existing microbial taxa without prior enrichment or target-specific amplification. Clinically, this method has demonstrated robust utility in *Orthoflavivirus* diagnostics. In a cohort of 112 dengue patients, Yadav *et al*. [157] used metatranscriptomic sequencing of blood to detect DENV RNA and simultaneously profile host and microbial transcripts, uncovering associations between microbial diversity and disease severity [157]. Similarly, a separate study using serum from DENV patients identified transcriptionally active microbial communities that differed by viral load, highlighting potential interactions between microbiota and immune responses [158]. Another recent study showed high-quality reconstruction of the ZIKV genome directly from clinical material using Ion Torrent metatranscriptomics and simultaneously detected novel viral sequences, emphasizing the technique’s diagnostic precision [159]. Beyond DENV and ZIKV, metatranscriptomic approaches have enabled the direct detection of WNV [160], JEV [160] and TBEV [161], from CSF and serum samples, demonstrating their broad clinical applicability across neurotropic orthoflaviviruses.

### Clinical application of viral metatranscriptomic sequencing

**Fig. 4.**
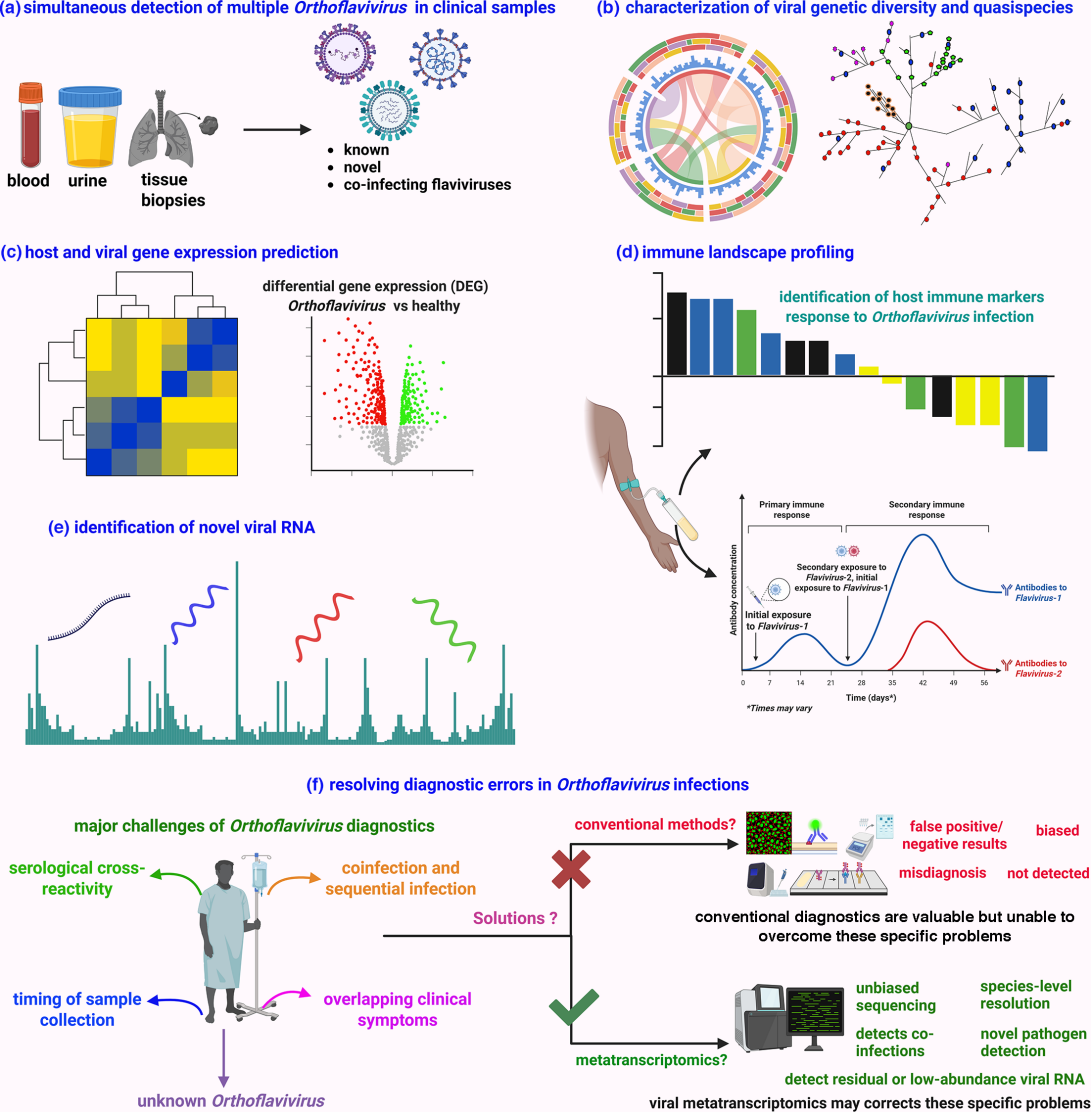
Clinical applications of metatranscriptomic sequencing in *Orthoflavivirus* diagnostics. (**a**) Simultaneous detection of multiple orthoflaviviruses in clinical samples, enabling comprehensive identification of co-infections or misdiagnosed cases without prior knowledge of the infecting agent. (**b**) Characterization of viral genetic diversity and intra-host quasispecies, providing insights into viral evolution, adaptation and potential drug resistance. (**c**) Profiling of viral and host gene expression, allowing real-time assessment of viral replication dynamics and host response. (**d**) Immune landscape profiling through transcriptomic signatures of cytokines, interferon-stimulated genes and immune cell markers, offering markers of disease severity and prognosis. (**e**) Discovery of novel viral RNAs, supporting virus discovery and improving genomic annotation. (**f**) Comparative overview of diagnostic accuracy showing how metatranscriptomics resolves limitations of conventional methods demonstrating its potential for implementation in clinical settings. No original experimental data are shown; the figure is conceptual and illustrative. Created with BioRender (https://www.biorender.com/).

In addition to its demonstrated performance across diverse case studies, viral metatranscriptomic sequencing offers a range of clinically relevant insights that extend well beyond pathogen detection. Viral metatranscriptomic sequencing offers a comprehensive approach to the clinical diagnosis of *Orthoflavivirus* infections, addressing key limitations of conventional methods. Fig. 4 provides a conceptual overview of the clinical applications of metatranscriptomic sequencing in *Orthoflavivirus* diagnostics. All panels are infographic representations summarizing key applications, including multi-virus detection, intra-host viral diversity profiling, host response assessment, immune landscape analysis, novel virus discovery and comparative diagnostic insights. As shown in Fig. 4a, this technique enables the simultaneous detection of multiple flaviviruses from clinical specimens, which is particularly valuable in regions where co-circulating arboviruses with similar clinical features complicate diagnosis. Recent studies have demonstrated direct detection of DENV RNA alongside host and microbial transcripts [157]. This method can simultaneously capture the virome and host response directly from low-biomass samples [162]. Beyond *Orthoflavivirus* detection, this technology has also been used to simultaneously detect and characterize Lassa virus [163]. It has also been applied to comprehensive characterization of viral genetic diversity and quasispecies, providing critical insights into circulating strains, recombination events and antiviral resistance mutations with direct clinical relevance (Fig. 4b). Recent studies have demonstrated its broad utility: for example, it successfully recovered complete genomes of 24 flavivirus species [164], enabled detailed characterization of intra-host viral diversity in DENV patients [157], identified 17 RNA viruses, including TBEV with diverse genomic architectures and evolutionary signatures [165], and detected recombination in 245 arboviral samples from southeast Australia [91].

These sequencing approaches enable real-time assessment of viral gene expression and simultaneous profiling of host immune responses from the same clinical sample (Fig. 4c, d), offering a powerful tool for understanding infection dynamics. By capturing actively transcribed viral RNA, this approach enables dynamic monitoring of viral gene expression, including structural (e.g. envelope and capsid) and non-structural proteins (e.g. NS1 and NS5), providing insights into viral replication kinetics, tissue tropism and infection stage [158,166, 167]. Additionally, it simultaneously records host transcriptomic responses, allowing in-depth profiling of immune activation patterns such as upregulation of interferon-stimulated genes (e.g. IFIT1 and ISG15), pro-inflammatory cytokines (IL-6, TNF-α) and antibody-related transcripts [168,170]. This approach facilitates disease staging, severity prediction and biomarker discovery, which is particularly valuable for infections such as DENV or ZIKV, where clinical presentations are variable and conventional diagnostics may prove inadequate. These applications enhance our understanding of host–pathogen interactions and strengthen the diagnostic and surveillance capacity of metatranscriptomic sequencing platforms.

Moreover, this approach has revealed numerous previously undetected RNA viruses across clinical, environmental and vector-derived samples and also facilitates the identification of subgenomic RNAs, non-coding regulatory elements and ORFs that are often missed by DNA-based metagenomics (Fig. 4e) [14,157, 169, 171]. These newly identified transcripts can encode hypothetical proteins with unknown function, which may represent novel structural components, immune evasion factors or replication enzymes, offering clues to their potential role in pathogenesis or host interaction. Thus, metatranscriptomics not only enhances pathogen detection but also drives the functional characterization of emerging viral genes, supporting efforts in antiviral target discovery and viral classification. Together, these applications position metatranscriptomics as a transformative tool in the clinical and epidemiological landscape of *Orthoflavivirus* diagnosis.

Therefore, this approach can be clinically implemented to overcome the specific limitation of *Orthoflavivirus* diagnosis, which faces several critical challenges, including serological cross-reactivity among related viruses, the narrow diagnostic window dictated by the timing of sample collection, complications arising from co-infections and sequential infections and nonspecific clinical symptoms that overlap among different *Orthoflavivirus* diseases (Fig. 4f). This raises a critical question: can conventional diagnostic methods adequately address these complexities? Conventional diagnostic methods such as serological assays and RT-PCR, while standard tools in current practice, often yield false-positive or false-negative results, exhibit detection bias due to primer specificity and frequently lead to misdiagnoses, especially in regions where multiple orthoflaviviruses co-circulate. In this context, metatranscriptomic sequencing emerges as a powerful alternative. By enabling unbiased, hypothesis-free detection of actively transcribed viral RNA at species or strain-level resolution, it allows for the identification of co-infections, novel or unexpected pathogens and comprehensive profiling of host–pathogen interactions. As such, metatranscriptomics offers a transformative approach to overcome the diagnostic limitations inherent in traditional *Orthoflavivirus* detection methods (Fig. 4f).

## Global implementation of metagenomics and metatranscriptomics in viral clinical diagnostics

**Fig. 5.**
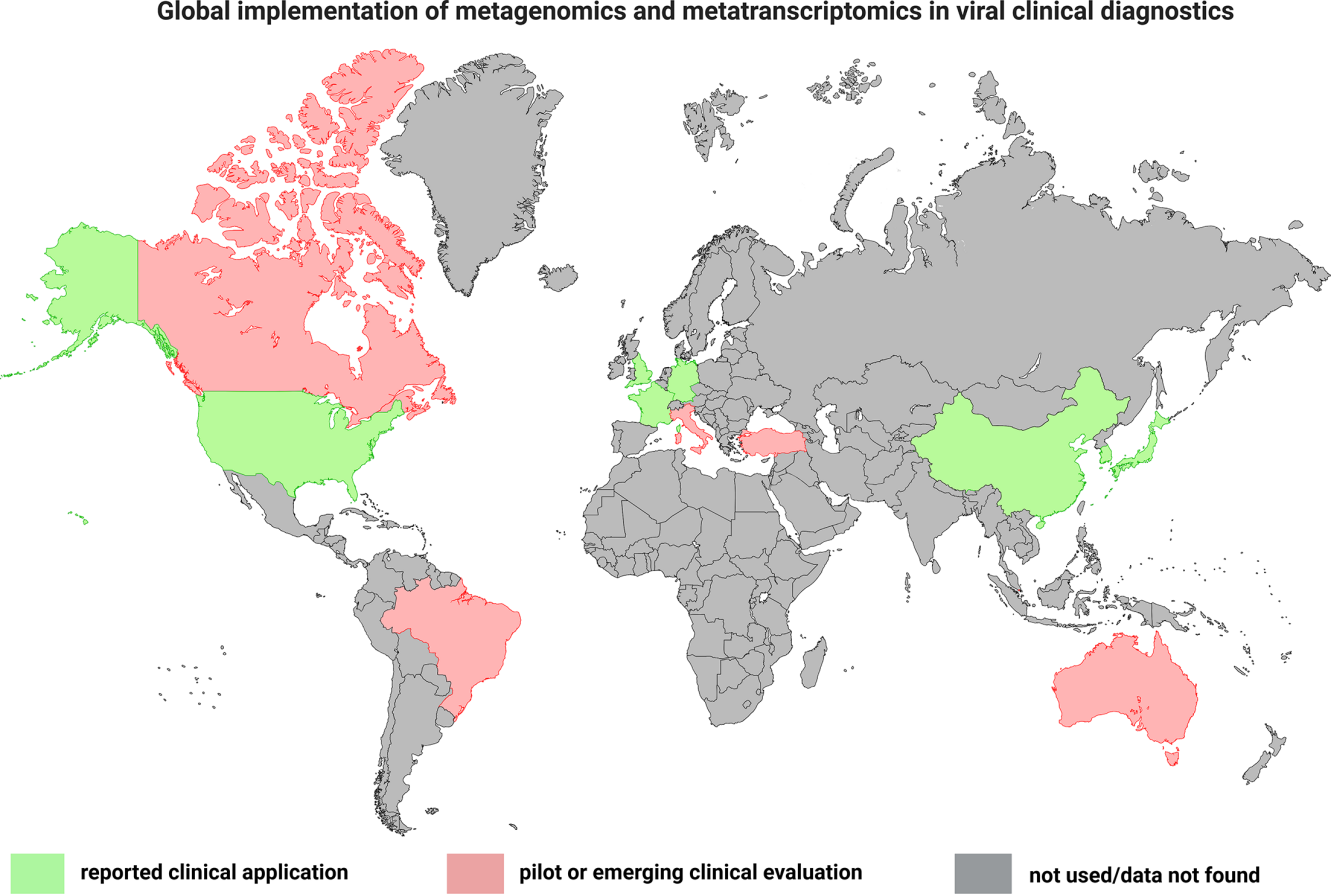
Global landscape of reported clinical applications of metagenomic and metatranscriptomic sequencing in viral clinical diagnostics. Colours indicate the level of published clinical evidence rather than healthcare system-wide adoption. Countries shown in green correspond to regions where metagenomic or metatranscriptomic sequencing has been actively applied in clinical diagnostic studies or reference laboratory workflows, as documented in peer-reviewed literature. Countries shown in red represent regions where evidence is currently limited to pilot, feasibility or proof-of-concept clinical studies. Countries shown in black indicate regions for which no peer-reviewed reports of clinical diagnostic application were identified or where data were unavailable at the time of writing. The map reflects published evidence of clinical application and does not imply nationwide implementation, routine diagnostic use or formal policy adoption. Created with BioRender (https://www.biorender.com/).

Originally developed in research-focused contexts, metagenomic and metatranscriptomic sequencing approaches are now increasingly being adopted in specialized clinical and reference laboratory settings to improve viral diagnostics, particularly in cases where conventional methods yield inconclusive or misleading results. Published clinical studies and institutional reports have demonstrated the translational potential of these technologies in real-world clinical practice. For example, clinical applications have been reported from the University of California, San Francisco (UCSF) Medical Center (USA) [22]; Guy’s and St Thomas’ Hospital and the UK Health Security Agency [19,24]; the Institut Pasteur (France) [20]; and leading hospitals across South Korea and Japan [172], China [23] and Germany [21], which have successfully integrated unbiased sequencing for pathogen detection, particularly in the diagnosis of encephalitis, respiratory syndromes, febrile illness and unexplained viral infections. Importantly, these reports reflect institution-level implementation rather than nationwide or routine adoption across healthcare systems. However, their widespread adoption remains limited. Emerging programmes in Canada, Brazil, Italy, Turkey and Australia are evaluating their clinical application through pilot studies, feasibility assessments or research-clinical interface programmes evaluating diagnostic utility (Fig. 5). The colour categories in Fig. 5 reflect the presence of published clinical studies rather than routine or system-wide diagnostic implementation. The metagenomics in healthcare market, valued at USD 1.5 billion in 2024, is projected to expand rapidly, reaching USD 5.9 billion by 2033, with a compound annual growth rate of 16.8% between 2026 and 2033. However, robust laboratory validation is essential for translating metagenomic and metatranscriptomics sequencing technology into reliable clinical diagnostics. For instance, Tan *et al*. [173] conducted a comprehensive validation of an mNGS assay targeting respiratory viruses, demonstrating sensitivity exceeding 95% and specificity greater than 98% across more than 300 clinical specimens, including challenging samples with low viral loads [173]. The assay exhibited strong reproducibility and robustness against potential interfering substances, supporting its potential suitability for routine diagnostic workflows following appropriate regulatory and laboratory validation. These comprehensive validation results provide a clear benchmark for *Orthoflavivirus* diagnostics, supporting the feasibility of implementing metagenomic sequencing in clinical settings.

## Future prospects

The integration of viral metagenomic and metatranscriptomic sequencing into clinical diagnostics for *Orthoflavivirus* infections holds transformative potential for infectious disease management, particularly in the context of emerging outbreaks, vaccine evaluation and global surveillance. However, to fully realize this promise, several critical and interconnected challenges must be strategically addressed. A foremost priority is the standardization of protocols across the entire diagnostic workflow, from sample collection and nucleic acid extraction to sequencing and bioinformatics. Variability at each step continues to hamper reproducibility and cross-study comparability. Encouragingly, recent advances such as commercially available, quality-controlled kits (e.g. NEB, Qiagen and Illumina) are helping to reduce inconsistencies. Further progress depends on establishing universal reference standards and quality assurance metrics to gain regulatory approval and foster clinical trust. Yet, technical barriers alone are not the primary bottleneck in many regions where orthoflaviviruses are endemic. The successful implementation of sequencing-based diagnostics in outbreak-prone and low-resource areas faces significant logistical and infrastructural constraints. These include limited access to trained bioinformatics personnel, unstable power supplies, slow or unreliable internet connectivity, inadequate local data storage infrastructure and the substantial cost of reagents, maintenance and data processing. Even portable technologies such as nanopore-based sequencing (e.g. MinION), CRISPR-mediated viral enrichment and lyophilized reagents, while promising, still require stable environmental conditions and technical support to function reliably in clinical contexts. Moreover, the complexity of sequencing outputs remains a challenge. Converting raw data into clinically actionable insights requires robust, validated bioinformatics pipelines, which is rarely feasible without sustained investment in human capacity and expertise. Platforms such as IDseq, CZID, One Codex and PathoScope are making strides towards user-friendly solutions, but meaningful interpretation still hinges on local expertise, contextual pathogen knowledge and clinical correlation. Therefore, capacity-building programmes in sequencing and bioinformatics tailored to local healthcare infrastructure are urgently needed to ensure sustainable implementation. Looking ahead, multi-omics integration linking metagenomics, metatranscriptomics, host transcriptomics, proteomics and metabolomics offers a systems-level view of *Orthoflavivirus* pathogenesis that can revolutionize our understanding of disease progression, co-infections and atypical presentations. However, such integration is currently limited to well-funded research settings and may not be immediately translatable to routine diagnostics. Realistically, achieving global equity in sequencing-based diagnostics will require long-term investments in infrastructure, affordable reagent access, secure and scalable data storage and cross-sector policy frameworks to support ethical governance, open data sharing and rapid outbreak responsiveness. Public health systems must incorporate sequencing literacy, and clinicians and laboratorians must be empowered through training programmes. Importantly, economic models must consider the total cost of ownership, including data management, workforce development and system upkeep, to avoid dependence on unsustainable, externally driven efforts. In summary, while sequencing technologies hold great promise, their clinical utility in *Orthoflavivirus* diagnostics depends not only on technological advancement but also on realistic, context-sensitive strategies that prioritize sustainability, equity and resilience. With a clear-eyed approach to addressing these challenges, sequencing-based diagnostics can progressively transition from niche research tools to central pillars of global infectious disease management and preparedness.

## Conclusion

As *Orthoflavivirus* outbreaks continue to rise in both endemic and newly affected areas, the limitations of traditional diagnostic tools have become increasingly apparent. Misdiagnoses due to antigenic overlap, low viral load, overlapping clinical symptoms or inadequate sampling windows compromise clinical outcomes and obscure epidemiological understanding. Viral metagenomic and metatranscriptomic sequencing offer powerful alternatives that are capable of unbiased pathogen detection, improved sensitivity across sample types and simultaneous profiling of co-infections and host responses. Evidence from clinical studies demonstrates that these approaches not only outperform conventional tests in complex or ambiguous cases but also directly impact patient care by informing accurate and timely diagnosis. Their implementation, however, must be supported by standardized workflows, cost-effective platforms and robust bioinformatic pipelines. As sequencing technologies become more accessible and global health systems increasingly recognize the need for precision diagnostics, the integration of metagenomics and metatranscriptomics into clinical practice will be pivotal for combating *Orthoflavivirus* infections and improving outbreak preparedness. This review underscores the clinical utility, operational considerations and future potential of these next-generation diagnostic tools in the fight against orthoflaviviruses.

## Abbreviations

ABS: amplicon-based sequencing
BHK-21: baby hamster kidney-21 cells
CCS: circular consensus sequencing
CFT: complement fixation test
CHIKV: chikungunya virus
CSF: cerebrospinal fluid
DENV: dengue virus
DETECTR: DNA endonuclease-targeted CRISPR trans reporter
dPCR: digital PCR
E: envelope protein
ELISA: enzyme-linked immunosorbent assay
HI: haemagglutination inhibition
IFAs: immunofluorescence assays
IFIT1: interferon-induced protein with tetratricopeptide repeats 1
IHC: immunohistochemistry
ISG15: interferon-stimulated gene 15
JEV: Japanese encephalitis virus
LRS: long-read sequencing
MN: microneutralization
mNGS: metagenomic next-generation sequencing
MVS: microfluidics-based viral sequencing
NS1: non-structural protein 1
PRNT: plaque reduction neutralization test
qPCR: quantitative PCR
SCM: single-cell viral metagenomics
SHERLOCK: specific high-sensitivity enzymatic reporter unlocking
SMS: shotgun metagenomic sequencing
TBEV: tick-borne encephalitis virus
TVE: targeted viral enrichment
Vero: African green monkey kidney cells
WNV: West Nile virus
YFV: yellow fever virus
ZIKV: Zika virus
